# LONG-TERM ALCOHOL CONSUMPTION AND STABILITY OF DAILY WORKING MEMORY PERFORMANCE IN OLD AGE

**DOI:** 10.1093/geroni/igac059.011

**Published:** 2022-12-20

**Authors:** Oliver Schilling, Denis Gerstorf, Anna Jori Lücke, Martin Katzorreck

**Affiliations:** University of Heidelberg, Heidelberg, Baden-Wurttemberg, Germany; Humboldt Universität zu Berlin, Berlin, Berlin, Germany; Heidelberg University, Heidelberg, Baden-Wurttemberg, Germany; University of Leipzig, Leipzig, Sachsen, Germany

## Abstract

Mixed evidence of associations of alcohol consumption with cognitive aging suggested that low to moderate alcohol consumption predicts more favorable cognitive outcomes than abstinence, whereas higher consumption operates as risk factor for cognitive decline. Daily short-term fluctuations of cognitive performance have also been established as risk factor for subsequent cognitive decline. Bringing these two lines of research together, our study analyzed associations of long-term trajectories of alcohol consumption with ambulatory assessments (7 days, 6 beeps per day) of working memory (WM) performance in participants (N = 155, aged 66-69 and 86-89) followed-up from a long-term (>20 years) longitudinal aging study. Overall, the findings do not support the “risk-view”, because long-term alcohol consumption patterns were not found to be predictive of either individual levels or intra-individual momentary fluctuations of WM performance. Follow-up analyses will examine the combined effects of alcohol consumption with further risk factors, such as long-term declines in health.

